# A chronic rejection model and potential biomarkers for vascularized composite allotransplantation

**DOI:** 10.1371/journal.pone.0235266

**Published:** 2020-06-26

**Authors:** Flemming Puscz, Mehran Dadras, Alexander Dermietzel, Frank Jacobsen, Marcus Lehnhardt, Björn Behr, Tobias Hirsch, Maximilian Kueckelhaus

**Affiliations:** 1 Department of Plastic Surgery, Burn Centre, BG University Hospital Bergmannsheil, Bochum, Germany; 2 Clinic for Orthopedics and Trauma Surgery, University Hospital Bonn, Bonn, Germany; 3 Division of Plastic Surgery, Department of Trauma, Hand and Reconstructive Surgery, University Hospital Muenster, Muenster, Germany; 4 Institute of Musculoskeletal Medicine, University Hospital Muenster, Muenster, Germany; 5 Department of Plastic, Reconstructive and Aesthetic Surgery, Hand Surgery, Fachklinik Hornheide, Muenster, Germany; University of Kentucky, UNITED STATES

## Abstract

**Background:**

Chronic rejection remains the Achilles heel in vascularized composite allotransplantation. Animal models to specifically study chronic rejection in vascularized composite allotransplantation do not exist so far. However, there are established rat models to study chronic rejection in solid organ transplantation such as allogeneic transplantation between the rat strains Lewis and Fischer344. Thus, we initiated this study to investigate the applicability of hindlimb transplantation between these strains to imitate chronic rejection in vascularized composite allotransplantation and identify potential markers.

**Methods:**

Allogeneic hindlimb transplantation were performed between Lewis (recipient) and Fischer344 (donor) rats with either constant immunosuppression or a high dose immunosuppressive bolus only in case of acute skin rejections. Histology, immunohistochemistry, microarray and qPCR analysis were used to detect changes in skin and muscle at postoperative day 100.

**Results:**

We were able to demonstrate significant intimal proliferation, infiltration of CD68 and CD4 positive cells, up-regulation of inflammatory cytokines and initiation of muscular fibrosis in the chronic rejection group. Microarray analysis and subsequent qPCR identified CXC ligands 9–11 as potential markers of chronic rejection.

**Conclusions:**

The Fischer344 to Lewis hindlimb transplantation model may represent a new option to study chronic rejection in vascularized composite allotransplantation in an experimental setting. CXC ligands 9–11 deserve further research to investigate their role as chronic rejection markers.

## Introduction

Within the past 20 years, vascularized composite allotransplantation (VCA) has emerged as a new therapeutic option for severe defects in reconstructive surgery. In this relatively young field of transplantation, acute rejection (AR) is mainly understood and can roughly be divided into cell mediated rejection and antibody mediated rejection [1]. In contrast, chronic rejection (CR) in the setting of VCA is still poorly understood [2,3].

As the number of clinical VCA is constantly increasing, CR presents a major obstacle to implement this method as a reliable treatment option in standard clinical practice; in particular, as VCA is usually not a life-saving procedure. Therefore, valid experimental models for the assessment of CR are vital to uncover underlying mechanisms behind CR with potential links for treatment and predict long-term outcome of these life-changing surgeries. Given the lack of currently available studies, there is no standardized, well-established animal model to assess CR in VCA so far.

In solid organ transplantation (SOT), animal models for CR research are well-established and used regularly. CR animal models mainly derive from a close major histocompatibility complex (MHC) compatibility between donor and recipient, which leads to mild immunological response and slow graft-deterioration in terms of CR. The Fischer344-Lewis rat model represents a frequently utilized combination in CR research, particularly in kidney transplantation [4]. The inbred rat strains Lewis (LEW) and Fischer344 (F344) are considered haploidentical and differ only at minor-histocompatibility loci, which are non-MHC encoding [5]. Hence, kidneys from F344 transplanted to LEW remain functionally intact for more than 100 days without immunosuppression and present histopathological changes similar to CR [4].

As the orthotopic hindlimb transplantation between fully MHC mismatched rat strains with irregular application of immunosuppression is the only known approach to simulate CR in a rodent model for VCA [6], we decided to investigate if higher MHC compatibility may be a more favorable method. Therefore, we transformed the Fischer344-Lewis model from SOT into a VCA setup to perform the orthotopic hindlimb transplantation between LEW and F344 rats.

Preliminary studies showed that skin allografts transplanted from F344 to LEW are rejected in 9–12 days due to AR [7], which is triggered by the high skin immunogenicity. These findings coincide with clinical findings, where skin is usually the first tissue to be targeted by AR [8].

By initiating this study, we sought to investigate the applicability of the Fischer344-Lewis model for the assessment of CR in VCA and identify potential biomarkers.

## Materials and methods

### Experimental groups and study design

**Fig 1:**
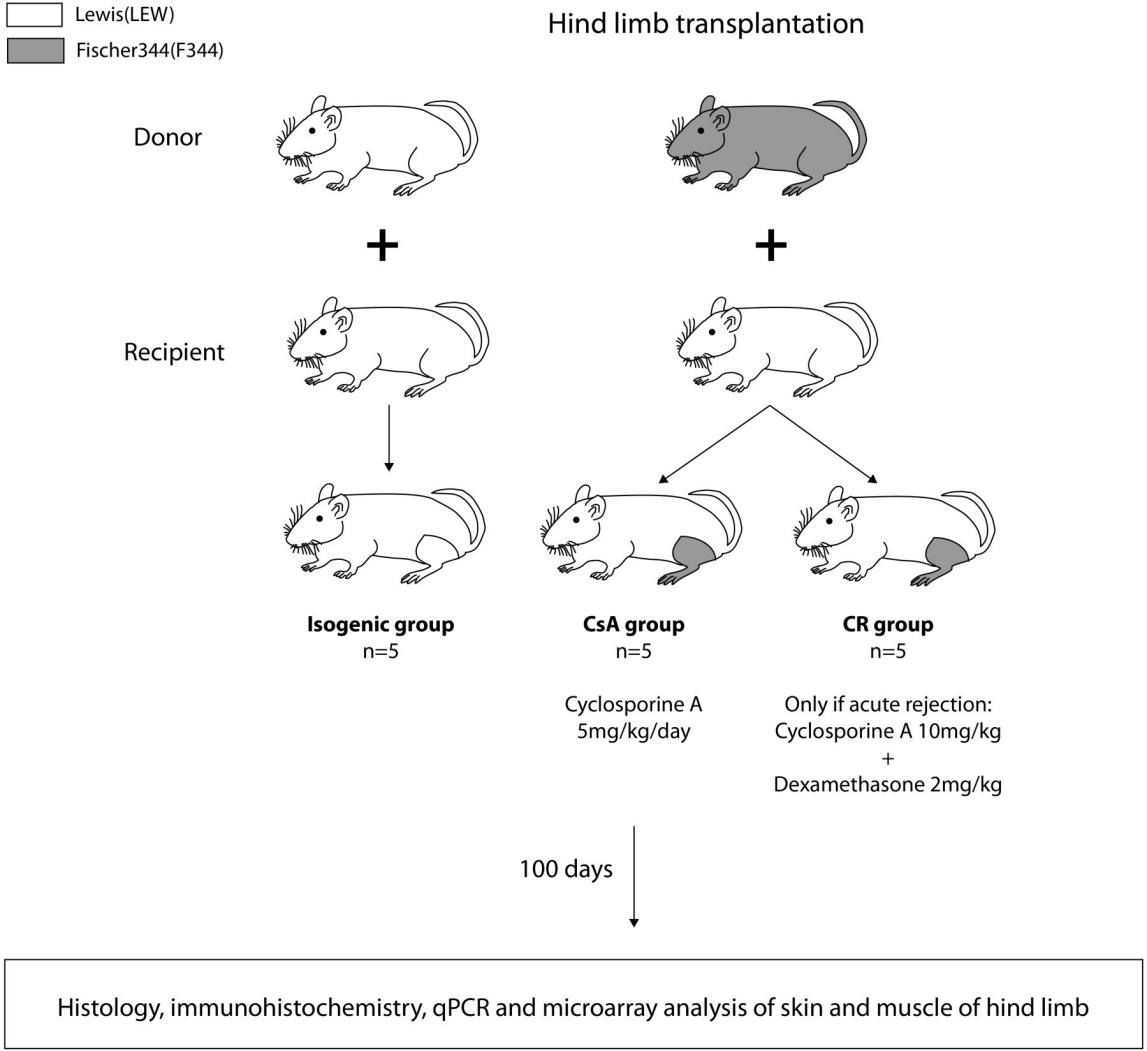
Group assignment and study design.

For group assignment of the transplantations confer Fig 1. Each group consisted of n = 5 animals. In all three transplantation groups, LEW served as recipients. F344-rats were donors in the CR group and the group receiving constant cyclosporine A (CsA group). All grafts were monitored daily for signs of rejection. The Iso group did not receive any immunosuppressive treatment. The CsA group received 5 mg/kg/day cyclosporine A via intraperitoneal injections. The CR group was treated only when signs of an acute skin rejection e.g. erythema or edema (defined as foot-swelling of ≥ 25%) occured. Treatment consisted of 10 mg/kg cyclosporine A and 2 mg/kg dexamethasone intraperitoneal and was administered daily until clinical signs of rejection completely disappeared.

Endpoints were either irreversible rejection (epidermolysis and/or necrosis of the graft) or postoperative day 100. At the endpoint, animals were euthanized by cervical dislocation under Isoflurane anesthesia and tissues were obtained for further analysis.

### Animals

All experiments were approved by the federal organization for animal care (LANUV NRW, registration number: AZ 84–02.04.2016.A330) and were performed in accordance with the European guidelines for laboratory animal care. Nine to ten week old female rats were purchased from Charles River, with a weight of 150–170 g (F344) and 190–210 g (LEW), respectively. They were maintained in an enriched, pathogen-free environment at our institution’s animal facility at a 12 h light/dark cycle and given access to standard laboratory chow and water ad libitum. Animals were held in groups of five in a European standard type IV cage (1800 cm^2^) with a height of 20 cm to enable standing at a constant room temperature of 22.5°C.

### Hindlimb-transplantation

Orthotopic hindlimb-transplantations were performed in a single-surgeon approach according to previous protocols [9,10]. In brief, the donor animal was anesthetized using Isoflurane 5% induction and 1–2% maintenance + 2 l/min O^2^. Analgesia was performed by subcutaneous injection of 0.1 mg/kg BW buprenorphine according to the guidelines of the German Society for Laboratory Animal Science.

After circumferential skin incision, femoral vessels, saphenous and sciatic nerve were dissected and transected proximally, to obtain sufficient length for vessel anastomosis and nerve coaptation. Amputation ended with muscle and femur transection. The femoral artery then was cannulated using a 27-gauge canula and the graft was flushed with 5 ml of cooled heparinized saline (50 IU/ml). The donor-limb was then stored at 4°C wrapped in wet gauze. The recipient’s limb was prepared in the same fashion. Limb rejoining began with the osteosynthesis, using an intramedullary rod, made from an 18-gauge needle. Femoral vessels and nerves were sutured using 10–0 nylon in an interrupted fashion. Ischemia time was 211 (95%CI: 203 min. to 219.2 min.) minutes on average. Muscle adaptation was performed, and transplantation ended with skin suture. Chinin-powder and spray-dressing were applied to the transplanted limb to prevent auto-mutilation.

### Histology and immunofluorescence

Muscle-, skin- and femoral artery-specimens were embedded in optimum cutting temperature compound (Tissue-Tek, Sakura Finetek Europe, Alphen aan den Rijn, Netherlands), snap frozen in isopentane and cut into 7 μm frozen sections. Microscopy was performed with Olympus IXplore pro (Olympus, Hamburg, Germany) and CellSens Imaging (Olympus, Hamburg, Germany).

For vasculopathy quantification, muscle and skin sections were stained with hematoxylin and eosin (for small vessel analysis) or Masson’s trichrome (for analysis of femoral arteries), respectively. Following this, we examined the sections at 10-fold (for femoral arteries) and 40-fold (for arterioles) magnification and measured vessel diameter, lumen diameter, intima thickness and media thickness. Intimal proliferation in femoral arteries was calculated with the formula(6):

$$\frac{Intimal\;thickness\;[\mu m]}{Intimal\;thickness\;[\mu m]+\;Media\;thickness\;[\mu m]}\;x\;100=Intimal\;proliferation\;[\%]$$

For arterioles in muscle and skin we used the occlusion ratio(6):

$$\left(\frac{Lumen\;diameter\;[\mu m]}{Vessel\;diameter\;[\mu m]}\;x\;100\right)-100=Occlusion\;ratio\;[\%]$$

Immunofluorescence staining was carried out on muscle 7 μm cryo-sections. Blocking was performed with 10% normal goat serum (AbCam, Cambridge, United Kingdom) in phosphate-buffered saline for one hour. All antibodies were used according to the manufacturer’s instructions. Briefly, slides were stained with primary antibodies (AbCam, Cambridge, United Kingdom): Anti-CD4 (ab203034), Anti-CD68 (ab125212) and Anti-Collagen3 (ab7778) + Anti-Sarcomeric Alpha Actinin (ab 9465), the last two performed as double-staining. After overnight incubation, secondary antibodies (ab6939 and ab150113, AbCam, Cambridge, United Kingdom) were applied and incubated for 1 hour. Sections were counterstained with DAPI (Thermo Fisher, Schwerte, Germany) and mounted with fluorescence mounting medium.

Three random sections for each specimen and three random areas per section for analysis were selected. All immunofluorescence images were procured at 20-fold magnification and analyzed using ImageJ-Software (National Institute of Health, Bethesda, MD, USA). Collagen 3 and actinin were quantified by counting positive stained pixels in relation to total pixel number and presented as a percentage. CD4 and CD68 were measured by counting positive stained cells in relation to total cell count and also presented as a percentage.

### Real time quantitative PCR

RNA was isolated from snap frozen muscle and skin samples using the RNeasy Fibrous Tissue Mini Kit (Qiagen, Hilden, Germany) and reverse-transcribed into cDNA by incubation with High-Capacity cDNA Reverse Transcription Kit with RNase Inhibitor (Thermo Fisher, Schwerte, Germany). Afterwards, the PCR reaction mix was prepared with TaqMan Universal Master Mix II, with UNG (Thermo Fisher, Schwerte, Germany) and 50 ng of cDNA per reaction. Real time qPCRs were performed with the StepOnePlus Real-Time PCR System (Thermo Fisher, Schwerte, Germany). Primers are listed in Table 1. Beta-Actin served as a house-keeping-gene. Each sample was tested in triplicate. Results were analyzed with the delta-delta Ct (ddCt) method [11], whereas mean and standard error of the mean were calculated from ddCt values. Exponentiates of these values were used for visualization as fold changes relative to untreated tissue on a log-scaled y-axis.

**Table 1:** TaqMan probes and assay IDs.

Target gene	Assay ID
IL6	Rn01410330_m1
TNF	Rn99999017_m1
IFNG	Rn00594078_m1
CXCL9	Rn00595504_m1
CXCL10	Rn01413889_g1
CXCL11	Rn00788261_g1
ACTB	Rn00667869_m1

### Microarray analysis

An amount of 100 ng of each RNA sample was hybridized to Agilent whole genome expression microarrays (G2514F, rat GE 4x44K v3, AMADID 028282, Agilent Technologies, Santa Clara, CA, USA). RNA labeling, hybridization and washing were carried out according to the manufacturer’s instructions. Images of hybridized microarrays were acquired with a DNA microarray scanner (Agilent G2505B) and features were extracted using the Agilent Feature Extraction image analysis software (AFE) version A.10.7.3.1 with default protocols and settings. The AFE algorithm generates a single intensity measure for each feature, referred to as the total gene signal, which was used for further data analyses using the GeneSpring GX software package version 14.9.1. AFE–total gene signals were normalized by the quantile method. Subsequently, data were filtered on normalized expression values. The gene expression data from our study have been deposited in the NCBI's Gene Expression Omnibus database (accession number: GSE133843).

For the identification of differentially expressed genes, only entities where at least 2 out of the total number of samples had values within the selected cut-off (50th - 100th percentile) were further included in the data analysis process. Using the GeneSpring GX software package version 14.9.1, differentially expressed genes were identified via moderated t-test (p-values < 0.05). Lastly, only mRNAs with a fold change ≥ 2.0 in the microarray analyses were further considered. Subsequent functional analysis of all genes overexpressed >4-fold in CR versus Iso muscle or skin was performed using the reactome database [12].

### Statistical analysis

Data was collected in Microsoft Excel 2017 (Microsoft, Redmond, WA, USA). Statistical analysis was carried out using GraphPad Prism 8.4.0 (GraphPad Software, La Jolla, CA, USA). Groups were compared with one-way analysis of variance (ANOVA) without assuming normal distribution (Kruskal-Wallis-ANOVA) and Dunn’s post-hoc test. All values were expressed as means with 95% confidence intervals (95%CI) or standard deviation (±) in brackets for n = 5 animals per group. A p-value < 0.05 was considered significant.

## Results

### Postoperative outcome and clinical appearance of hindlimbs

**Fig 2:**
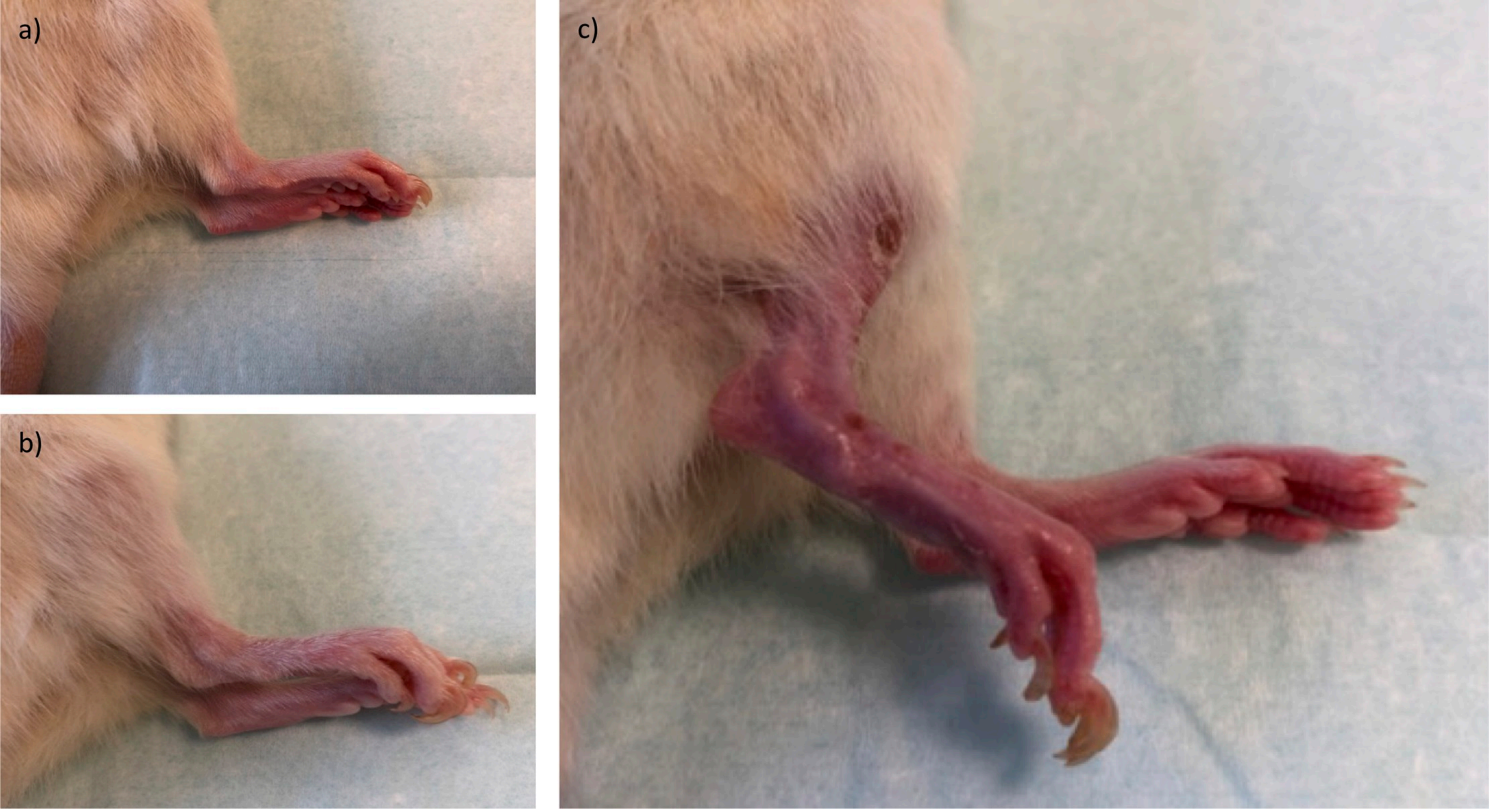
Hind limbs at POD 100. a+b) Iso and CsA group hind limbs did not present any clinical signs of inflammation or rejection. c) CR hind limb: Muscle and skin atrophy as well as hair loss is noted in comparison to native limb (background).

Postoperative, regular wound healing took place in all three groups. None of the hindlimbs presented vascular thrombosis and/or anastomotic leakage. At the endpoint, hind limbs in the Iso and CsA group showed non-irritated skin with regular display of adnexa. Representative photographs of Iso and CsA hindlimbs can be seen in Fig 2A and 2B. Animals in these groups used the transplanted hindlimb regularly to both stand and walk on it. Further analysis of motoric function was not performed.

Hindlimbs that underwent chronic rejection (CR group) presented morphologically normal at the start of observation and after first episodes of rejection. After several rejection episodes, slight erythema did not resolve completely even after 3 boli of cyclosporine and dexamethasone. All animals reached the 100-days endpoint, no episode of acute rejection was present at euthanasia.

At the 100-days endpoint, atrophy in muscles and skin as well as hair loss was seen in the CR group only (Fig 2C). Furthermore, CR group animals did not show any motor function in the transplanted hindlimb.

### Frequency of acute skin rejection episodes

Isogenic transplanted and CsA hindlimbs did not show any clinical signs of acute skin rejection (e.g. erythema, edema) throughout the follow-up time. CR group hindlimbs underwent an average of 7 (± 3.9) acute skin rejection episodes within the 100 days of follow-up. Symptoms of acute rejection resolved mainly after 1–3 boli of cyclosporine A and dexamethasone. The slow progressive loss of skin appendages, dermal sclerosis and muscular atrophy could not be resolved.

### Vasculopathy

**Fig 3:**
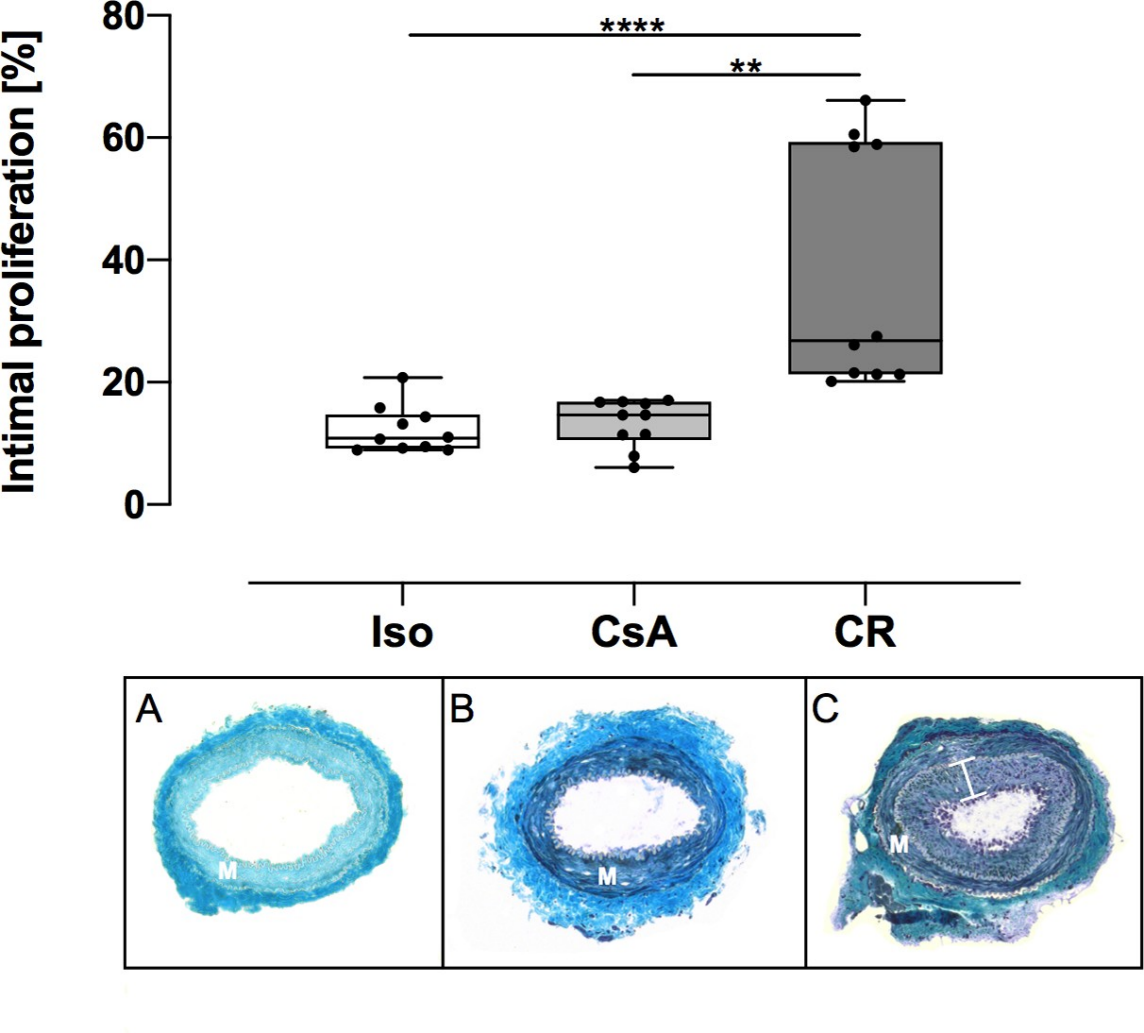
Intimial proliferation in femoral arteries at POD 100 in percent. Above: Data is presented as individual data points and boxplots; boxplots show the median, 25%- and 75%-quartile; whiskers indicate the minimum and maximum. In the CR group, the femoral artery showed a significantly elevated intimal proliferation rate in comparison to Iso and CsA group, as indicated (**: *p* < 0.01; ****: *p* < 0.0001). Below: Representative images of Iso (A), CsA (B) and CR (C) group, Masson's trichrome stain at 10x. Intimal proliferation and luminal occlusion is seen in the femoral artery of the CR group (M: media; bracket indicates the CR group intima).

Femoral arteries in the Iso and CsA group did not show relevant intimal hyperplasia. The average intimal proliferation in the Iso group was 12.25% (95%CI: 9.5% to 15%) and 13.35% (95%CI: 10.52% to 16.18%) in the CsA group, respectively. Distinct increase in intimal proliferation was seen in the CR group (Fig 3). On average, intimal proliferation here was 38.21% (95%CI: 23.99% to 52.43%) and thus significantly higher than in the Iso (*p* < 0.0001) or CsA (*p* = 0.0012) group (Fig 3).

Arterioles in CR muscle (mean: 62.23%, 95%CI: 55.16% to 69.3%) were significantly narrowed in comparison to Iso (mean: 34.13%, 95%CI: 27.35% to 40.91%, *p* < 0.0001) and CsA muscles (mean: 39.3%, 95%CI: 33.37% to 45.22%, *p* < 0.0005).

**Fig 4:**
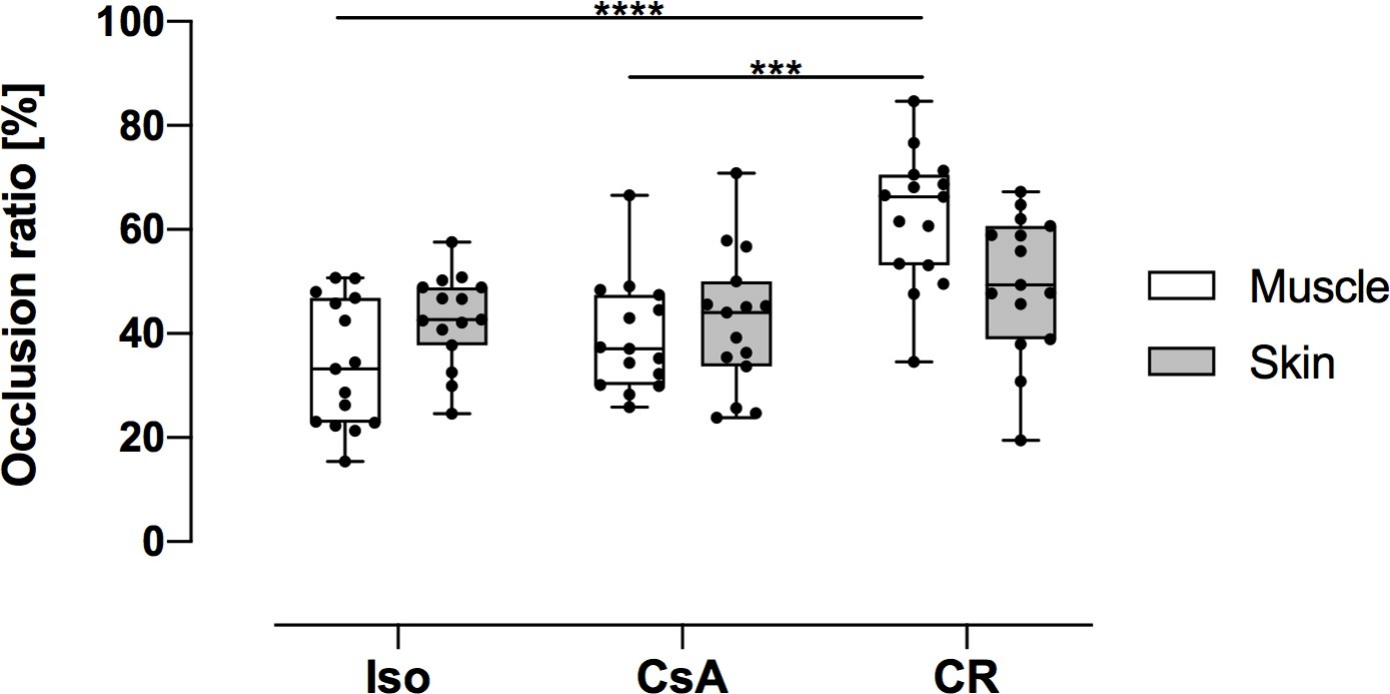
Arterioles occlusion ratios in muscle and skin at POD 100. Data is presented as individual data points and boxplots; boxplots show the median, 25%- and 75%-quartile; whiskers indicate the minimum and maximum. CR group arterioles in muscle tissue demonstrated a significantly higher occlusion ratio in comparison to Iso and CsA groups (***: *p* < 0.001; ****: *p* < 0.0001).

Arterioles in the skin also showed a trend towards differences in occlusion ratios, yet these results did not reach statistical significance (*p* = 0.21 and 0.12) (Fig 4).

### CD4- and CD68-positive cellular infiltration

**Fig 5:**
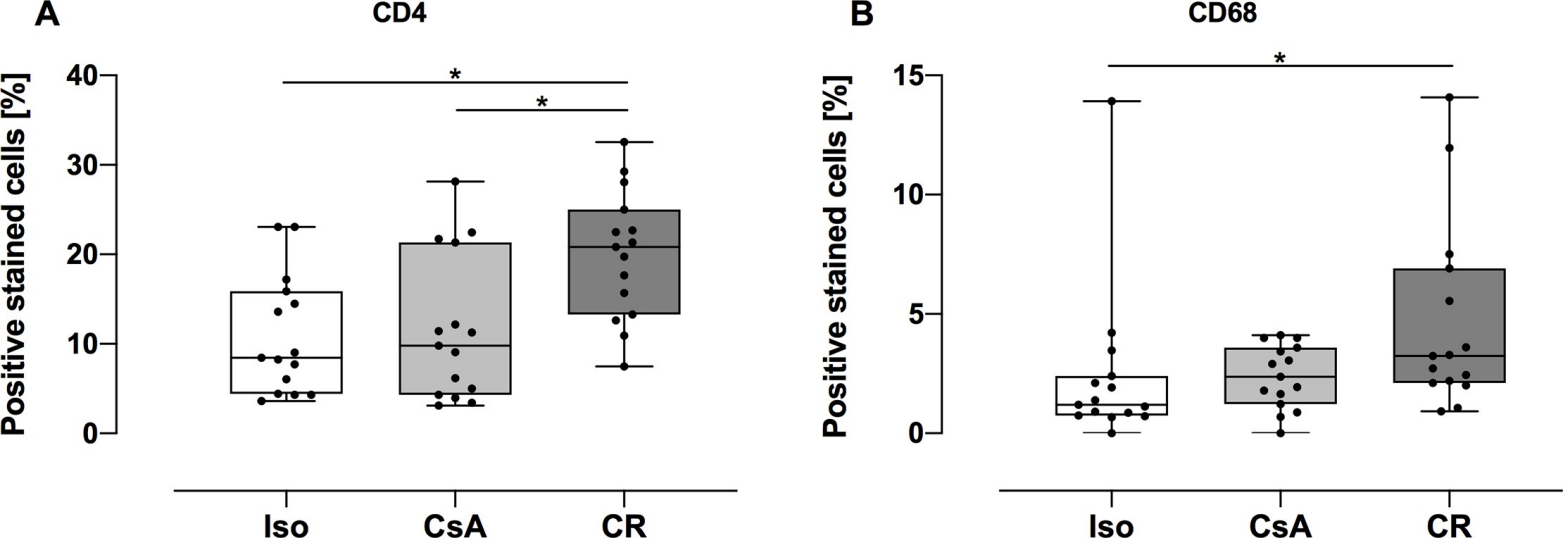
CD4 and CD68 positive stained cells in muscle tissue at POD 100. Data is presented as individual data points and boxplots; boxplots show the median, 25%- and 75%-quartile; whiskers indicate the minimum and maximum. Number of CD4 positive cells was significantly elevated in CR group in comparison to Iso and CsA groups. Count of CD68-positive stained cells revealed significantly higher cell count in CR group in comparison to the Iso muscle (*: *p* < 0.05).

The number of CD4-positive cells in muscle tissue was significantly increased in the CR group (mean: 19.97%, 95%CI: 16.02% to 23.92%) compared to Iso (mean: 10.9%, 95%CI: 7.24% to 14.56%, *p* = 0.0113) and CsA (mean: 11.56%, 95%CI: 7.06% to 16.05%, *p* = 0.0109) groups (Fig 5A).

The relative number of CD68 positive cells was significantly higher in CR muscle (mean: 4.64%, 95%CI: 2.46% to 6.82%) compared to muscle of Iso group (mean: 2.38%, 95%CI: 0.5% to 4.25%, *p* = 0.0149) but not to muscle of CsA group (mean: 2.37%, 95%CI: 1.64% to 3.11%, *p* = 0.2701) (Fig 5B).

### Muscular fibrosis

**Fig 6:**
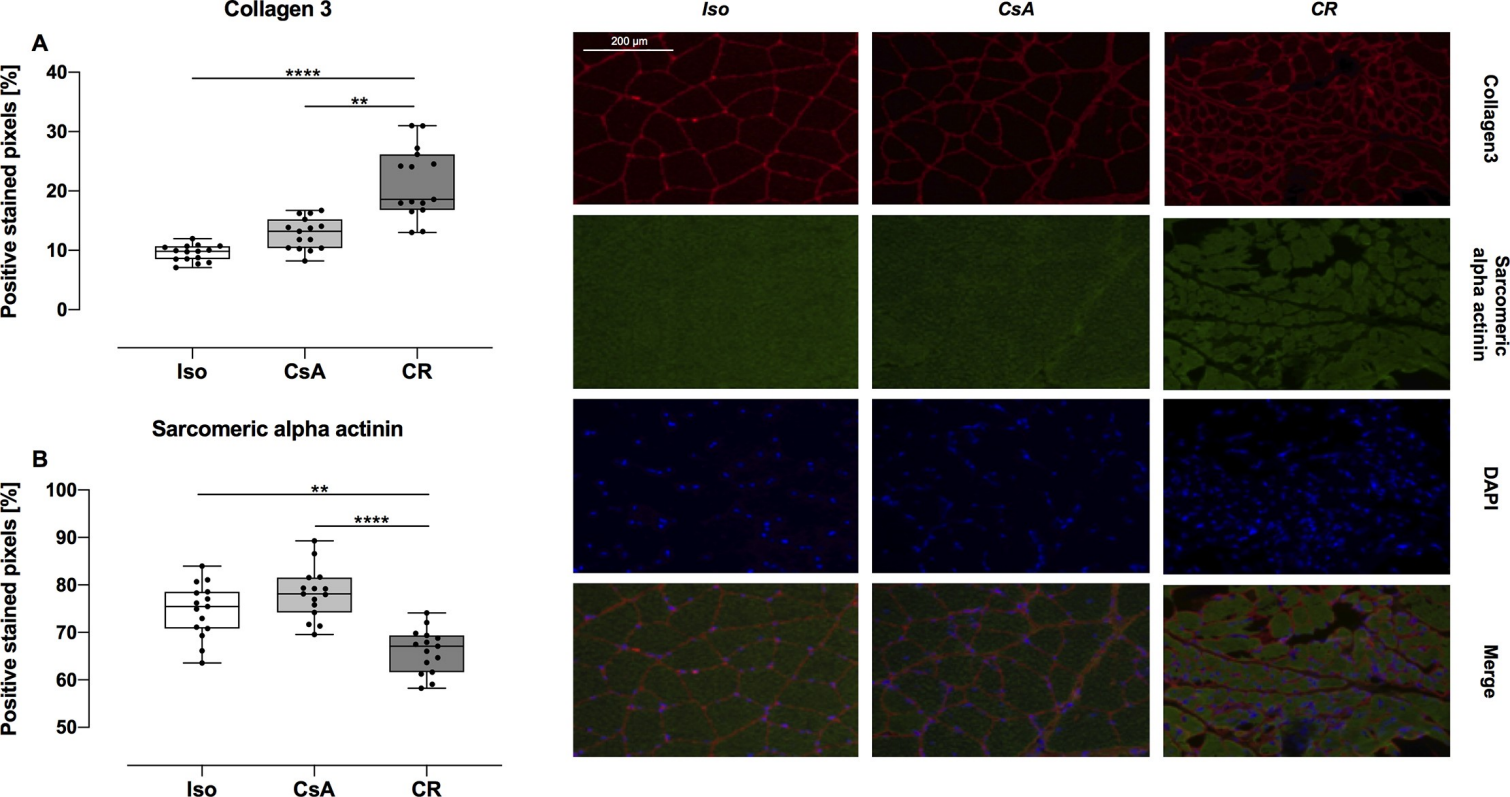
Amount of collagen3 (A) and sarcomeric alpha actinin (B) in muscle at POD 100. Data is presented as individual data points and boxplots; boxplots show the median, 25%- and 75%-quartile; whiskers indicate the minimum and maximum. CR muscle showed a significant increase in collagen 3, whereas sarcomeric alpha actinin is significantly decreased, compared to Iso and CsA muscle. Representative immunofluorescent images of Iso, CsA and CR muscle tissue (**: *p* < 0.01; ****: *p* < 0.0001).

Results from double-stained muscle tissue are displayed in Fig 6A and 6B. The relative amount of sarcomeric alpha actinin was significantly lower with 66.08% (95%CI: 63.52% to 68.65%) in the CR group compared to 74.67% (95%CI: 71.51% to 77.83%, *p* = 0.002) and 78.16% (95%CI: 75.17% to 81.14%, *p* < 0.0001) in the Iso and CsA groups, respectively. Collagen 3 was significantly elevated in the CR group (mean: 21.36%, 95%CI: 18.09% to 24.64%) compared with Iso (mean: 9.53%, 95%CI: 8.77% to 10.29%, *p* < 0.0001) and CsA (mean: 12.81%, 95%CI: 11.35% to 14.28%, *p* = 0.0032) muscle.

### Gene expression

**Fig 7:**
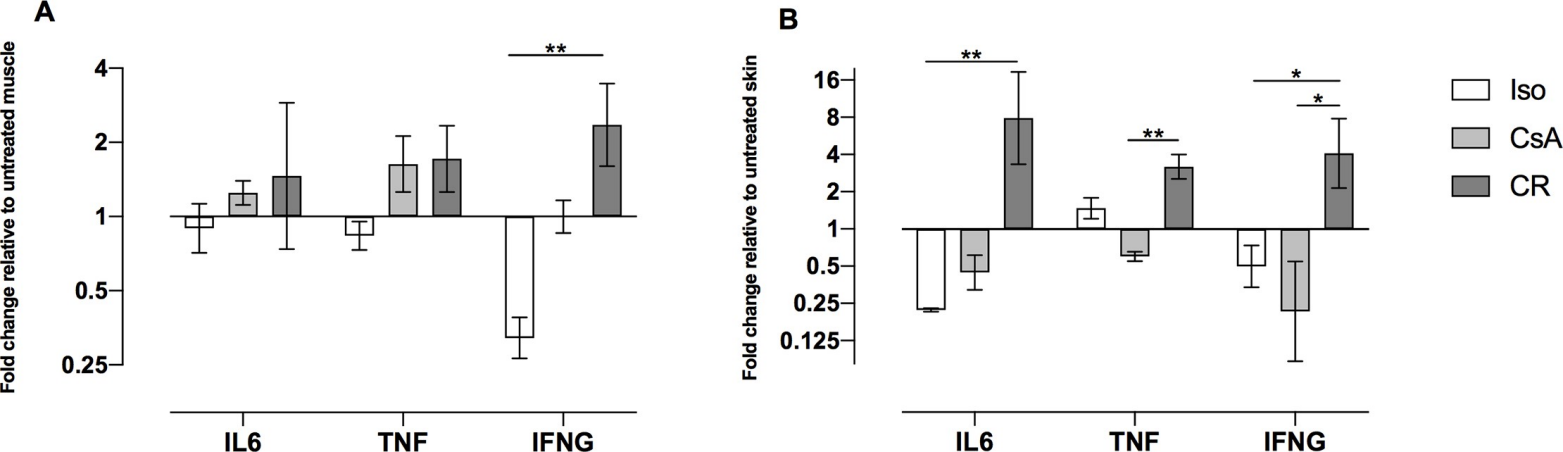
A+B: mRNA expression of *Interleukin 6* (IL6), *Tumor necrosis factor* (TNF) and *Interferon gamma* (IFNG) in muscle (A) and in skin (B), expressed as fold changes relative to untreated tissue. Bar graphs indicate the fold change (2^-ddCt^) and standard error of the mean on a log-scaled y-axis. Significance was tested using dCt values. Mean and standard error of the mean were calculated from ddCt values. Although significance was not reached for all targets, results show that pro-inflammatory cytokines were highly elevated in the CR group (*: *p* < 0.05; **: *p* < 0.01).

Real time pPCR results are shown in Fig 7A+7B. In CR muscle tissue, pro-inflammatory cytokines *Interleukin 6* (IL6), *Tumor necrosis factor* (TNF) and *Interferon gamma (*IFNG*)* were elevated in comparison to Iso and CsA muscle although statistical significance could only be found for IFNG (*p* = 0.0037). Similar trends could be observed in CR skin with statistical significant upregulation of IL6 compared to Iso skin (*p* = 0.0075) and TNF compared to CsA skin (*p* = 0.0018). IFNG showed significant upregulation compared to both Iso (*p* = 0.0473) and CsA skin (*p* = 0.0324).

**Fig 8:**
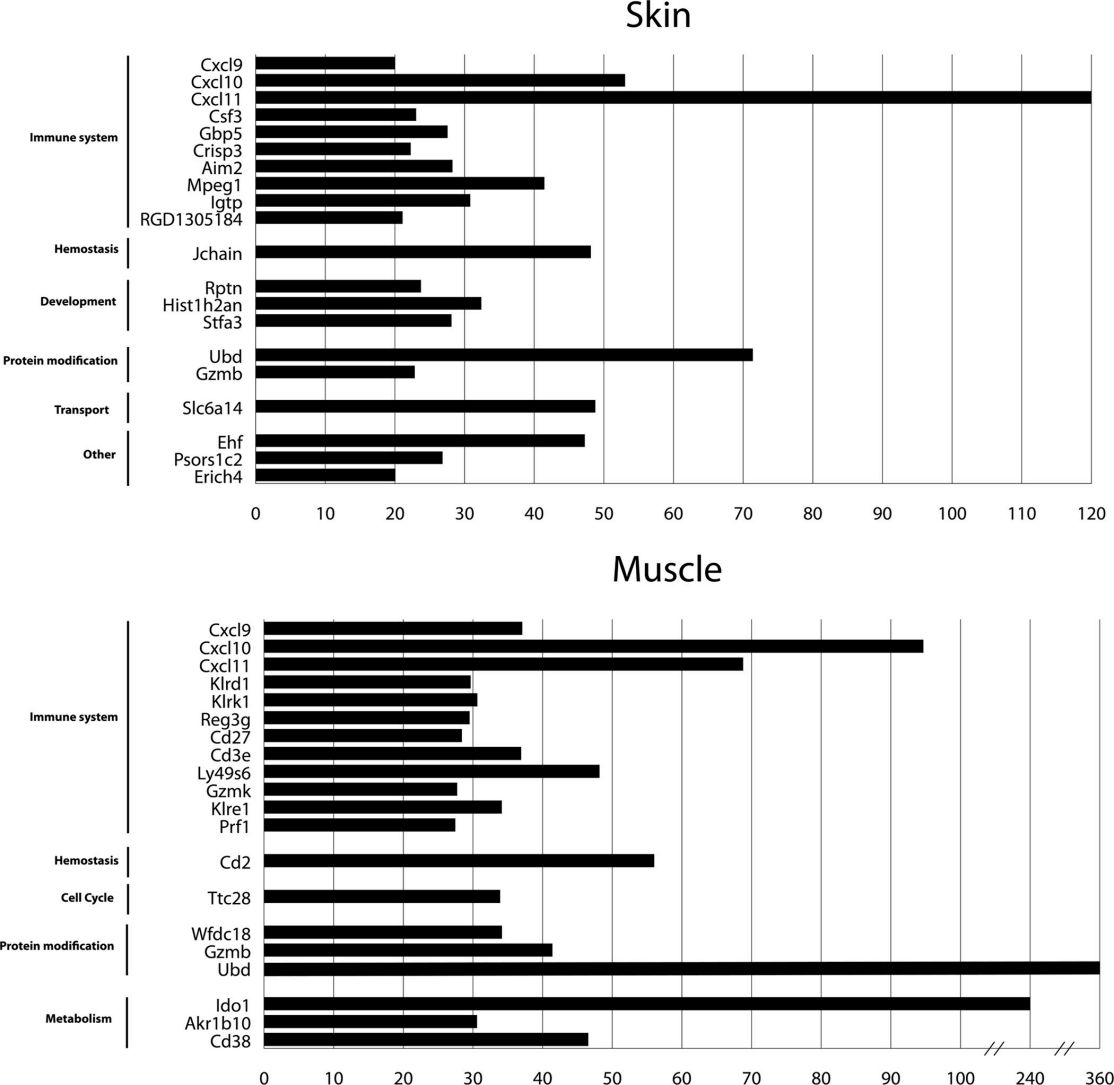
Genes with highest fold change in expression levels of CR versus Iso group upon Microarray analysis.

Exploratory microarray gene expression analysis was performed with 2 samples per group for skin and muscle. Gene expression of CR versus Iso skin and muscle were compared. Fig 8 shows the 20 genes, which had the highest fold changes in skin and muscle tissue. In both tissues, most of these genes had functional roles in the immune system including CXC ligand (CXCL) 9, CXC ligand 10 and CXC ligand 11 as players of interferon signaling.

**Fig 9:**
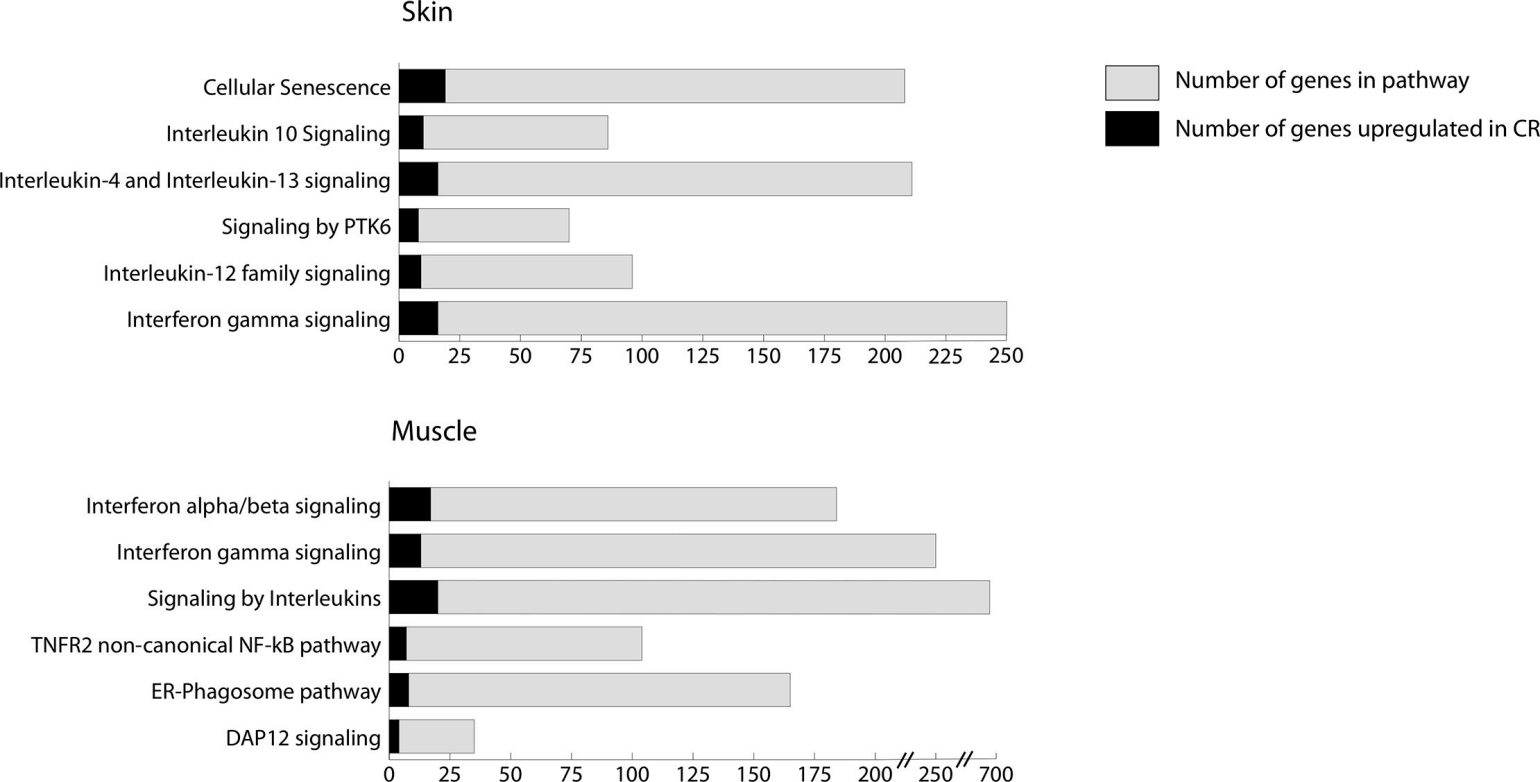
Reactome overrepresentation pathway analysis of genes with fold change ≥ 4.

Subsequent functional analysis of all genes overexpressed >4-fold in CR versus Iso muscle or skin was performed using the reactome database. Fig 9 shows the pathways most overrepresented in statistical order. Interferon signaling was amongst the highest overrepresented in both muscle and skin tissue.

**Fig 10:**
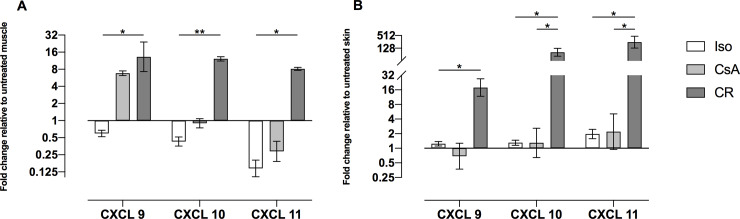
A+B: mRNA expression of CXC ligand (CXCL) 9, CXC ligand 10 and CXC ligand 11 in muscle (A) and in skin (B), expressed as fold changes relative to untreated tissue. Bar graphs indicate the fold change (2^-ddCt^) and standard error of the mean on a log-scaled y-axis. Significance was tested using dCt values. Mean and standard error of the mean were calculated from ddCt values. Both CR muscle (A) and skin (B) did show significant elevations of these chemokines, as indicated (*: *p* < 0.05; **: *p* < 0.01).

For further analysis, qPCR analysis for CXCL 9, 10 and 11 were performed. Results are displayed in Fig 10A and 10B. All three chemokines were significantly upregulated in CR muscle and skin in comparison with Iso- or CsA-group.

## Discussion

The purpose of this study was to validate the orthotopic hindlimb transplantation between the rat strains LEW and F344 concerning its feasibility and potential benefits in assessing CR in VCA.

We were able to induce and detect significant intimal proliferation in the CR group. Besides allograft-vasculopathy, we could also show the immigration of immunological relevant cells, such as CD4- and CD68-positive-cells but also their deteriorating effects on muscle tissue. In terms of gene expression, we demonstrated the up-regulation of specific inflammatory cytokines and highlighted interferon signaling.

In clinical VCA, insufficient immunosuppression is likely to be responsible for the above mentioned long-term changes [13–15]. Thus far, irregular application of immunosuppression has been the only approach to imitate CR in an experimental setup. Whether or not this simulation is accurate remains debatable [16]. Clinical observations reported that patients with allogeneic transplanted faces undergo 1–3 acute skin rejection episodes in the first year after transplantation [17]. Preliminary animal studies that produced characteristics of CR in VCA used completely MHC mismatched rat-strains for the assessment of CR in VCA (e.g. Brown Norway to Lewis) and quoted 19 (±3.2) acute skin rejection episodes in a group that received irregular immunosuppression during 90 days of follow up [6]. The high number of AR episodes was able to induce changes, e.g. intimal proliferation, sclerotic skin, muscular fibrosis and up-regulation of pro-inflammatory pathways, which have been associated with CR [6]. However, in the clinical setting chronic rejection has occurred after longer time spans post transplantation with fewer acute rejection episodes, suggesting graft remodeling without presence of acute rejection. Therefore, our experimental model mimics the clinical scenario more accurately.

Due to small donor pools and low tolerance towards ischemia, MHC matching has not been given the highest priority for VCA-procedures. In contrast, in SOT, higher MHC-compatibility is linked to better graft function, longer graft survival and reduction of immunosuppressive treatments with fewer rejection episodes [18]. A recent study by Ng et al. [19] analyzed the outcomes of 20 face and upper extremity transplantations in burn patients. Although they could not demonstrate correlations between the MHC-matching status and the occurrence of AR episodes, less intensive treatment of AR episodes was necessary in patients with “better” MHC match. In contrast, Berglund et al. [20] did find a significant correlation between MHC class II mismatch and incidence of AR and development of donor-specific-antibodies (DSA) [21]. Our results, concerning the number of AR may be a hint towards a positive correlation between MHC matching status and number of AR episodes, nevertheless a direct comparison between this high MHC-compatible and a complete mismatched MHC model may bring more clarity concerning the role of MHC matching in VCA.

Clinical appearance at post-operative day 100 revealed sclerotic skin, loss of adnexae, atrophy and crusted ulcerations on the graft. These findings mirror the clinical appearance of CR in VCA [3].

Muscle fibrosis, defined as replacement of functional muscle tissue by stromal elements, has already been reported in VCA [6,16]. However, muscle tissue is rather difficult to obtain in the clinical setting. We detected decreased levels of sarcomeric alpha actinin, acting as a marker for viable muscle tissue and elevated Collagen 3 levels in the CR group, indicating that muscular fibrosis took place in CR group muscle. Inaccurate nerve adaptation with impairment of motor function and aftermath of ischemia reperfusion injury may be possible bias causing loss of muscle tissue. Compared to the Iso and CsA groups, muscle tissue in the CR group did show decreased sarcomeric alpha actinin levels in immunofluorescent images, and therefore can be considered as an independent process in the CR group.

DAPI staining exposed cellular infiltration in CR muscle. Therefore, we decided to quantify both CD4 and CD68 positive cells, as these cell types are known to be major participants in the development of tissue remodeling processes occuring during CR [22]. CD4 positive cells, mostly referred to as CD4 T helper cells, play a crucial role in the development of CR by synthesis and elaboration of a series of cytokines that evoke effector mechanisms of graft rejection [23]. CD68 positive cells represent the downstream inflammatory cascade. CD4 positive cells are one of the major producers of IFNG [24], which is known to be a potent cytokine and chemoattractant for macrophages [25]. Macrophages, which in turn are attracted into the tissue secrete cytokines and growth factors, finally leading to extracellular matrix deposition and metaplastic processes [26]. Our results confirm these propositions and underline the importance of CD4 positive cells in CR reactions. Furthermore, we confirmed preliminary studies, who could also demonstrate CD4 and CD68 positive cell immigration [6,27,28]. However, CD68 only reached significant difference between the CR and the Iso group. Yet, results from Collagen 3 and sarcomeric alpha actinin staining confirm the likely fibrotic effects of these cell lines on muscle tissue.

Graft vasculopathy appears to be the common thread when it comes to CR in VCA. Hence, it was reported in most cases of CR in VCA [29]. Intimal proliferation, leading to vascular occlusion, ischemia and finally resulting in necrosis of the graft, represent the most important findings [30]. In this study, the femoral arteries in the CR group did show significant intimal proliferation, validating that we did successfully induce processes that lead to this consistent change within the graft. In contrast, findings by Mundiger et al. [31,32], who discovered significant intimal proliferation in a nonhuman primate VCA-model, suggest that intimal proliferation may occur even in the absence of AR.

Increased occlusion ratio in smaller vessels did also confirm our findings, especially in CR muscle tissue, where we achieved highly significant results.

In a clinical setting, diagnosis of intimal proliferation poses a challenge. Novel diagnostic tools e.g. ultrasound biomicroscopy [33] may be promising non-invasive diagnostic approaches.

Cytokines play an important role in CR development. In this study IL6, TNF and IFNG showed increased levels in CR skin and muscle.

IL6 plays a critical role in mediation of cell-mediated rejection, antibody-mediated rejection and graft vasculopathy in kidney transplantation [34]. Jordan et al. have discussed the high importance of IL6 during kidney rejection and moreover, highlighted possible treatment options by using IL6 inhibitors (e.g. tocilizumab) successfully [34].

Previous works also detected elevated levels of IL6 in experimental VCA models [27,28], but to our knowledge, a specific correlation between IL6 elevation and CR has not been shown and IL6-inhibitors have not yet been applied in VCA. Besides IL6, TNF is also a potent cytokine and nuclear factor κB dependent, which finally leads to T-cell development and proliferation [35]. Elevated TNF expression could be observed in muscle of hindlimbs undergoing rejection, which was also described in previous experimental VCA rejection models [28,36]. IFNG presents another major cytokine regulating inflammatory and rejection processes [37]. In human kidneys it has been shown to be a potent regulator of chronic rejection processes [38]. Few studies highlight the role of IFNG in VCA: Friedmann et al. [28] and Datta et al. [36] demonstrated up-regulation of IFNG in acute rejection and in ischemia reperfusion injury respectively.

We demonstrated the up-regulation of IL6, TNF and IFNG, three multifunctional cytokines, in our CR model. Future research should focus on their roles as possible bio-markers or even treatment targets.

Exploratory micro array analysis detected upregulated genes, that have not been described in relation to CR in VCA before, although statistical significance of the results could not be achieved due to the low sample size (n = 2).

Ubd, encoding for the protein Ubiquitin d showed upregulation in both skin and muscle. Although Famulski et al. [39] already described a relation between Ubiquitin d and IFNG in rejected mouse kidneys, there is no further literature concerning this topic. Therefore, the role of Ubiquitin d in VCA remains to be determined.

In addition to the up-regulation of IFNG seen in qPCR, exploratory microarray analysis pointed to upregulated downstream genes, such as CXC ligand 9, 10 and 11. These ligands are mostly produced by macrophages after IFNG interaction. They showed high fold changes in skin and muscle of the CR group. For further validation, we performed qPCRs and demonstrated their significant increase in skin and muscle of the CR group. CXCL 9, 10 and 11 belong to the CXC chemokine family interacting with the chemokine receptor CXCR3, which mainly regulates immune cell migration, differentiation, and activation [40] Consistent to our findings, Krezdorn et al. recently found elevated CXCL 11 in chronically rejected facial allografts [41]. Interestingly, in their study CXCL 11 is elevated both in faces during acute and chronic rejection. Urinary CXCL 9 and 10 have both been identified as potential biomarkers of subclinical rejection in kidney transplants [42]. For this purpose, CXCL 9 was also validated in a multicenter clinical study [43]. An association of serum CXCL 9 levels with acute rejection of liver allografts has been shown [44].

Therefore, serum and tissue levels of the CXCL proteins may be an interesting biomarker in VCA-patients undergoing chronic rejection.

This study has limitations that should be considered: in contrast to acute rejection, the term chronic rejection does not impose clear diagnostic criteria but represents a multitude of changes in allografts that mediate long-term deterioration. Our approach to simulate chronic rejection, similar to that of other studies, consisted of repeated limited acute rejection episodes to ultimately result in changes similar to chronic rejection. The presence of true chronic rejection as observed clinically can therefore be challenged.

Moreover, we have to point out that animal models themselves have limitations and can never be transferred 1:1 into the clinical setting, but due to few clinical cases, these models may be a more accurate way to obtain novel hints in VCA research.

## Conclusion

We were successfully able to demonstrate the hindlimb transplantation between Fischer344 and Lewis. Moreover, we were able to induce changes known to be specific for chronic rejections, e.g. intimal proliferation by permitted episodes of limited acute rejection. The closer histocompatibility of the utilized species resulted in a lower frequency of acute rejection episodes compared to reports in fully mismatched models. In microarray and subsequent qPCR analysis we identified the CXCR3 ligands CXCL 9, CXCL 10 and CXCL 11 as potential biomarkers of chronic rejection in VCA.

Therefore, this model may provide a new platform to gain further insight into chronic rejection in VCA.

Overall, further research in many fields of VCA is required to enable a standing as a reliable clinical option in reconstructive surgery.

## Supporting information

S1 Checklist (PDF)

S1 Dataset (XLSX)

S2 Dataset (XLSX)

S3 Dataset (XLSX)

S4 Dataset (XLSX)
